# Technical note—the use of an inclinometer to accurately guide intra-operative femoral de-rotational osteotomies

**DOI:** 10.1093/jhps/hnad021

**Published:** 2023-07-31

**Authors:** Alistair Iw Mayne, Owen J Diamond

**Affiliations:** Musgrave Park Hospital, Stockman’s Lane, Belfast BT9 7JB, United Kingdom; Musgrave Park Hospital, Stockman’s Lane, Belfast BT9 7JB, United Kingdom

## Abstract

Femoral de-rotational osteotomies are a safe and effective treatment for symptomatic excessive femoral anteversion or retroversion. The author’s preferred technique for performing a de-rotational osteotomy is via a subtrochanteric transverse osteotomy with intramedullary nail fixation. We describe a method for guiding femoral de-rotation correction intra-operatively using a bubble inclinometer.

## INTRODUCTION

Femoral de-rotational osteotomies are a safe and effective treatment for symptomatic excessive femoral anteversion or retroversion [1, 2]. Normal femoral version is defined as the angular difference between the axis of the femoral neck and the transcondylar axis of the knee [3]. Abnormal femoral version can occur in isolation or alongside other pathological hip pathology including dysplasia and femoro-acetabular impingement. Pathological rotational deformities can result in abnormal hip biomechanics, resulting in potential instability as well as impingement, with damage to the hip articular cartilage and labrum.

Following a detailed history, clinical examination and review of radiographs, patients should undergo three dimensional radiological imaging with CT or MRI scan of the femur performed to accurately measure their femoral version. MRI has been shown to be as accurate as CT for measuring femoral version, and avoids ionizing radiation, and is therefore our preferred option [4]. A wide range of normal values for femoral anteversion have been reported in the literature, but most would consider normal anteversion to lie between 10 and 25 degrees [5]. The degree of correction angle is based on the patient’s gait (which we formally assess through a gait lab analysis), clinical examination of rotational profile in prone and supine positions, and their radiological measurement compared to normal values. The aim is to correct to within normal radiological values, provided this will not leave the patient with an extreme clinical abnormality, and therefore careful clinical examination and patient counselling is essential.

For patients with symptomatic abnormal femoral version, a number of proximal, diaphyseal and distal femoral osteotomies have been described. The author’s preferred technique for performing a de-rotational osteotomy is via a subtrochanteric transverse diaphyseal osteotomy approximately 5 cm distal to the lesser trochanter, stabilized with intramedullary nail fixation.

## SURGICAL TECHNIQUE

The senior author’s preferred device is a commercially available analogue bubble inclinometer (Lev-O-Gage Inclinometer) which has been found to be more intuitive and easier to use compared to a digital inclinometer which only lists positive values. The inclinometer is placed in a sterile transparent arthroscopy camera bag by the scrub team during operating room set-up. This is not a medical device and we describe its use solely as an additional adjunct/check for hip surgeons to help aide surgical accuracy.

Following anaesthesia, the patient is positioned supine on a traction table with the ipsilateral foot placed in a foot holder and the contralateral leg placed in a leg holder in an abducted and externally rotated position.

Following skin preparation and draping, the femur is initially prepared for insertion of a long intramedullary nail with guidewire insertion and sequential reaming of the intramedullary canal performed under image intensifier guidance. This is performed prior to the osteotomy and deformity correction. Once the chosen nail length and diameter have been determined, the nail is assembled ready for insertion. The nail diameter is normally 1 mm less than the reamer diameter when there is initial cortical ‘chatter’—this is to allow the nail to pass following derotation but also means the nail is large enough to give adequate stability to encourage union. Two 3.2 mm percutaneous threaded guidewires are then inserted (larger diameter wires are less likely to bend in the soft tissue). The proximal wire is placed at the level of the distal aspect of the greater trochanter where the bone begins to flare into the trochanter, in a posterior position to allow reamer/nail passage. Nail entry point is at the junction of the anterior third and posterior two thirds of the greater trochanter. The distal wire is inserted posteriorly at the level of the femoral condyles, again to ensure there is room for reamer/nail passage. It is important to take care to ensure the guidewire is bi-cortical for stability and that the position is not going to interfere with nail insertion (i.e. guidewires are placed posteriorly to intramedullary canal/nail). The guidewires are inserted parallel to the floor of the operating theatre. At this point, the inclinometer is placed on the guidewires to measure the angle of wire insertion relative to the horizontal plane and these angles are recorded. The wires do not have to be perfectly parallel to the floor as it is the change in angle that is important for calculating the correction. The initial angles of the proximal and distal wires prior to osteotomy are then recorded on a whiteboard in the operating theatre.

**Fig. 1.**
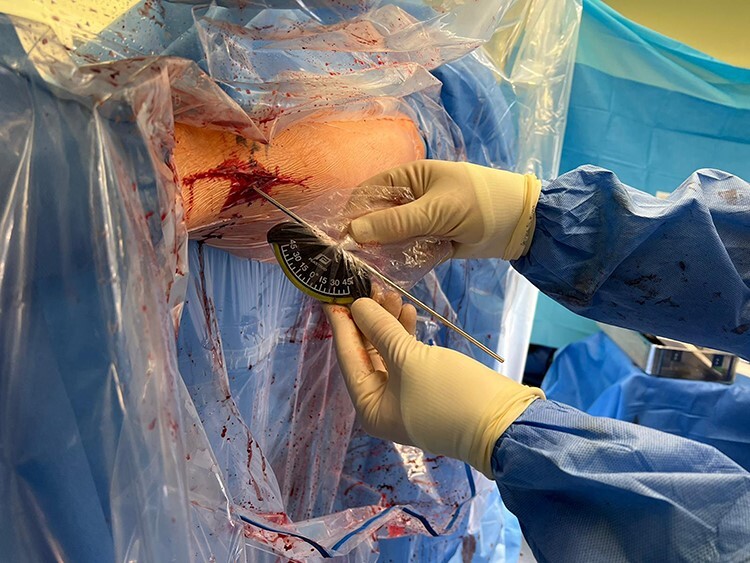
Angle of distal wire relative to horizontal being measured after osteotomy with bubble inclinometer.

**Fig. 2.**
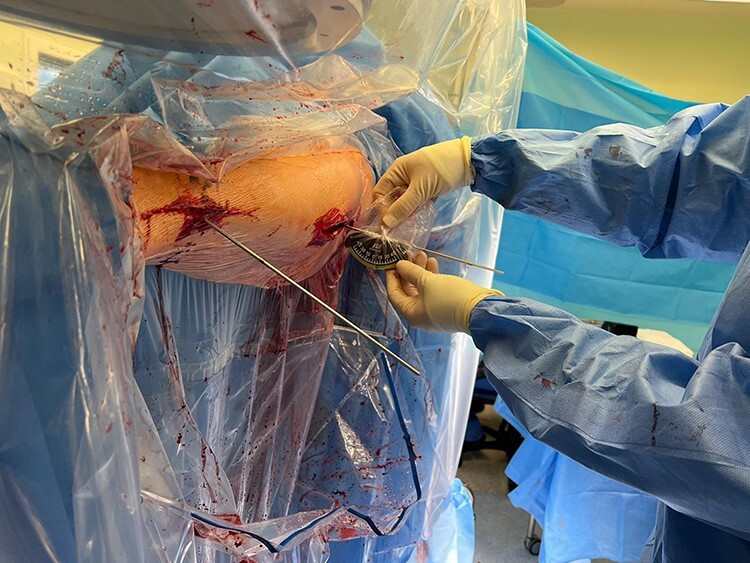
Angle of proximal wire relative to horizontal being verified following osteotomy.

After confirming the correct level on the femur using an image intensifier, a lateral subvastus approach is made at the subtrochanteric level. Care is taken to minimize excessive soft tissue and periosteum disruption. Broad Hohman retractors are placed anteriorly and posteriorly to the femur at the osteotomy level to protect the adjacent soft tissues. Two large bone holding forceps are placed on the femur just proximal and distal to the planned osteotomy site and these are key to controlling and correcting the derotation following osteotomy. At this point, the antegrade femoral intramedullary nail is inserted under image intensifier control over the guidewire to just proximal to the planned osteotomy site, and the intramedullary guidewire is retracted to the same level. An oscillating saw is then used to create a transverse osteotomy. Following completion of the osteotomy, the femur is provisionally de-rotated using the bone holding forceps and the antegrade nail is passed across the osteotomy site to give provisional stability. The amount of femoral de-rotation is now accurately determined by using the bubble inclinometer on each of the two guidewires previously inserted (Figs. 1 and 2). The difference in angle between the two wires will equate to the amount of femoral de-rotation (assuming both wires were parallel prior to the osteotomy or alternatively measurements can be made based on pre-osteotomy inclinometer readings). If further correction is required, then the nail can be backed out slightly and further correction achieved with the bone holding forceps.

The nail is then fully inserted, locked proximally and fracture site compressed, before a final confirmation of de-rotation correction using the inclinometer is made and the nail is locked distally. The osteotomy site is visualized to confirm good bony apposition. An image intensifier is used to confirm final nail position.

## DISCUSSION

The role for correction for abnormalities of femoral version is increasingly recognized within the hip preservation community and technical accuracy is important to ensure optimized patient outcomes. There is widespread variation in the surgical technique and accurate correction can be technically challenging. An inclinometer is an instrument used for measuring angles of slope (or tilt) of an object relative to gravity’s direction. Previous research from our unit has demonstrated improved accuracy of total hip arthroplasty acetabular component operative and radiological inclination when an inclinometer is used to guide component insertion [6, 7]. The use of inclinometers to guide femoral derotational osteotomies has also been described by Stiebel and Paley [8]. Furthermore, a sawbone study by Swarup et al. has confirmed the high surgical accuracy of femoral de-rotation when guided by a digital electronic inclinometer [9]. We have found a bubble inclinometer to be more intuitive and easier to use compared to a digital inclinometer which only lists positive values.

## Summary

Femoral de-rotational osteotomy is a common procedure for correction of pathological femoral version. We have described the use of a bubble inclinometer to help guide accurate on-table correction of pathological femoral version.

## DATA AVAIILABILITY

There are no new data associated with this article.
